# Analysis of Calcium Sulfate Scaling Phenomena on Reverse Osmosis Membranes by Scaling-Based Flux Model

**DOI:** 10.3390/membranes12090894

**Published:** 2022-09-17

**Authors:** Fumio Yokoyama, Mitsutoshi Nakajima, Sosaku Ichikawa

**Affiliations:** 1 Faculty of Life and Environmental Sciences, University of Tsukuba, Tsukuba 305-8572, Japan; 2 Alliance for Research on Mediterranean and North Africa, University of Tsukuba, Tsukuba 305-8572, Japan

**Keywords:** reverse osmosis membrane, calcium sulfate, mass-transfer coefficient, scaling-based flux model, scaling-based critical flux, scaling index

## Abstract

In this study, the behavior of permeate flux decline due to scale precipitation of calcium sulfate on reverse osmosis membranes was investigated. The proposed scaling-based flux model is able to explain that permeate fluxes attributed to three mechanisms of scale precipitation—cake formation, surface blockage, and mixed crystallization—converge to the same newly defined scaling-based critical flux. In addition, a scaling index is defined, which determines whether scale precipitates on the membrane. The experimental results were analyzed based on this index. The mass-transfer coefficients of flat membrane cells used in the experiments were measured and, although the coefficients differed, they could be summarized in the same form as the Leveque equation. Considering the results of the scale precipitation experiments, where the operating conditions of pressure, solute concentration, temperature, and Reynolds number were varied, the convergent values of the permeate fluxes are explained by the scaling-based critical fluxes and the scale precipitation zones by the scaling indexes.

## 1. Introduction

Since Loab and Sourirajan developed a workable reverse osmosis (RO) membrane of cellulose acetate in 1960 [1], the development of the RO process over the past six decades has been remarkable. A major application of the RO process is the production of drinking water by seawater desalination, including the Ashkelon desalination plant that processes 330,000 m^3^/d, equivalent to about 15% of the domestic water consumption in Israel, and desalination applications for drinking water and industrial water production by brackish water desalination [2]. The RO process is an indispensable technology for the production of ultrapure water for the cleaning of semiconductors and liquid crystals. In regions that have domestic water shortage areas, such as the U.S., China, India, the Middle East, and Singapore, the RO process is used to produce drinking water, industrial water, and agricultural water using municipal sewage as raw water. Factory wastewater is treated by the RO process and reused as industrial water. In the food industry, the RO process is widely used for the concentration and recovery of valuable materials, such as the concentration of juice from oranges and tomatoes, and recovery of whey in the dairy industry [3,4].

The main problem in the practical operation of RO plants is the prevention and prediction of RO membrane performance degradation. The causes of performance degradation of RO membranes can be divided into two categories: degradation of the RO membrane itself, including chemical degradation due to oxidation and hydrolysis, biological, and physical degradation; and fouling of the RO membrane, which includes the adsorption of dissolved substances, deposition of organic and colloidal materials, and precipitation of sparingly soluble inorganic substances (scale) on the membrane. Of these, the precipitation of sparingly soluble inorganic substances, such as calcium, magnesium, and silica compounds, on RO membranes is a problem in brackish water desalination, causing restriction of the recovery ratio of product water. In seawater desalination, the problem is the increasing risk of precipitation of calcium and magnesium compounds in the concentrated brine due to the increasing recovery ratio of the produced water [5].

Okazaki and Kimura [6] were the first to analyze scale precipitation phenomena on RO membranes from a chemical engineering point of view. They proposed the ‘cake formation’ mechanism, in which precipitated scale forms a cake layer, to explain the permeate flux decline due to scale precipitation and investigated the transient state of permeate flux decline in detail, including a study of the waiting period for scale precipitation to start from the supersaturated state of solute concentration. However, in the convergent stage of permeate flux decline, they only stated that the solute concentration at the membrane surface became equal to the saturated concentration and the permeate flux converged to a constant value. Regarding analyses of scale precipitation phenomena on RO membranes, many reports have been presented on experiments related to the transient state, in which scale precipitation occurred and the permeate flux decreased rapidly [7,8,9,10,11,12,13,14,15,16]. Experiments and analyses of the transient state of scale precipitation on RO membranes based on the cake formation mechanism were conducted by Okazaki and Kimura [6] and other researchers [8,17].

In contrast, a surface blockage mechanism was proposed by Gilron et al. [18] and was later followed by supporting reports [7,19,20,21,22,23,24,25]. Experiments and analyses based on this mechanism were performed by Gilron et al. and others [18,20,21,22,23,26]. In response to these competing theories, Lee et al. reported that precipitation was due to both surface blockage and cake formation occurring during precipitation, based on experimental data [27]. Their view was subsequently supported by experimental observation and analyses by other researchers [9,10,28,29,30,31,32,33,34,35,36,37].

The convergent stage of permeate flux decline due to fouling on the membranes in RO, nanofiltration, ultrafiltration, and microfiltration was intensively investigated based on three indices regarding the deposition of organic and colloidal suspended materials on the membranes: critical flux, below which no fouling occurs and a decline of flux with time does not occur [38,39,40,41,42,43,44,45,46,47,48,49,50,51,52,53,54,55,56]; threshold flux, at or below which a low or nearly constant rate of fouling occurs and above which the rate of fouling increases markedly [44,46,49,50,52,53,54,55,56,57,58,59]; and limiting flux, which represents the maximum stationary permeate flux that can be reached by increasing the transmembrane pressure [42,44,46,49,55,60,61].

In contrast, there are only a few reports on the convergent stage of permeate flux decline during scale precipitation on RO membranes. Critical flux detection in a silica scaling RO system was studied by Lisitsin et al. [19]. The application of an equation for determining the critical flux of colloidal fouling due to scale precipitation was studied by Shirazi et al. [30]. These reports studied only the transient state of permeate flux decline or the proposed application of an analytical model of colloidal fouling (deposition) to scale precipitation on the membrane surface, so further studies are needed.

In this study, we focused on the convergent stage of the permeate flux decline at the time of precipitation of sparingly soluble inorganic substances (scale) on the RO membrane and investigated the operating conditions in the scaling (scale precipitation) zone, which is an important factor for RO membrane performance degradation in practical operation.

Here, *deposition* and *precipitation* are defined as follows: deposition is a phenomenon in which insoluble suspended materials in water adhere to the membrane surface; precipitation is a phenomenon in which solute dissolved in water is concentrated to become insoluble material that adheres to the membrane surface.

## 2. Materials and Methods

### 2.1. Reagents and Concentration Measurement Methods

The sodium chloride (NaCl) and calcium sulfate dihydrate (CaSO_4_·2H_2_O) used in this experiment were special-grade reagents from Fujifilm Wako Pure Chemical Corp., Osaka, Japan. Pure water (electrical conductivity less than 1.0 × 10^−4^ S/m) was obtained from tap water treated with an ion-exchange resin cartridge supplied by Nomura Micro Science Co., Ltd., Atsugi, Japan.

The solutions of NaCl and CaSO_4_ were prepared by dissolving NaCl and CaSO_4_ · 2H_2_O of the reagents in pure water. The solute concentrations of NaCl and CaSO_4_ were measured according to the following relationship between electrical conductivity and concentration, which was determined at 25 °C by preliminary investigation:

*Y* = 1522*X*^2^ + 4720*X*, (1)

where *X* is electrical conductivity (S/m), and *Y* is NaCl concentration (mg/L) within the range of less than 1000 mg/L.

The formula for the relationship between electrical conductivity and CaSO_4_ concentration at 25 °C is as follows:

*Y* = 15,200*X*^2^ + 6150*X*, (2)

where *X* is electrical conductivity (S/m), and *Y* is CaSO_4_ concentration (mg/L).

### 2.2. Reverse Osmosis Membranes

The four types of RO membranes used in the experiments were made of cross-linked fully aromatic polyamide composites and were manufactured by Toray Industries, Inc., Tokyo, Japan. Table 1 shows the performance datasheet information supplied by the membrane manufacturer.

### 2.3. Flat Membrane Cells

**Figure 1:**
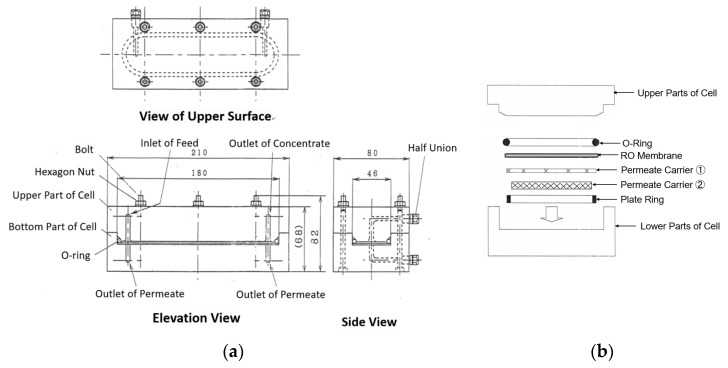
Flat membrane cell. (**a**) External view, (**b**) Inside structure.

Cells A and B used in the experiments were Membrane Master CT-10 lamellar flow-type flat membrane test cells of transparent acrylic resin made by Nitto Denko Corp., Osaka, Japan. The effective membrane area was 58.4 cm^2^ and the cross-sectional area of the feed channel was a rectangle with a length of 0.75 mm and width of 36.0 mm. External and internal views of the cell are shown in Figure 1a,b, respectively.

### 2.4. Experimental Equipment

**Figure 2:**
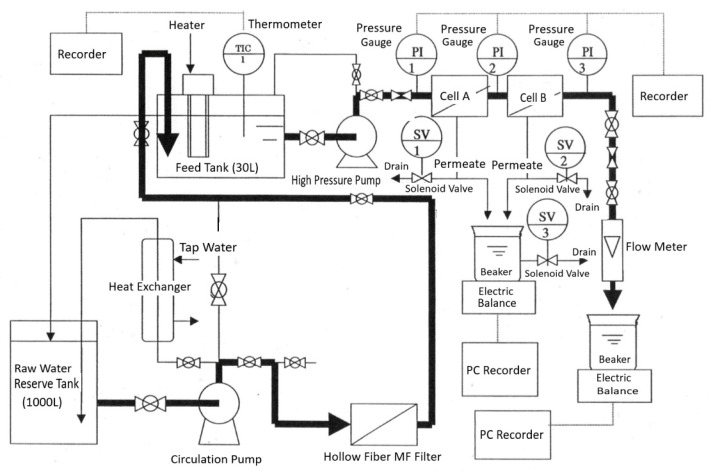
Flow diagram of experimental equipment.

A flow diagram of the experimental equipment used for the mass-transfer coefficient measurement and scale precipitation experiments is shown in Figure 2. In the mass-transfer coefficient measurement, NaCl and CaSO_4_ were used as solutes; in the precipitation experiments, CaSO_4_ was used as the solute. The concentration of NaCl or CaSO_4_ was adjusted in the raw water reserve tank (capacity: 1000 L). Raw water was supplied to a feed tank with a capacity of 30 L by a circulation pump. Some of the raw water that overflowed the feed tank was returned to the raw water reserve tank. A polysulfone hollow fiber filter (Toraycube, manufactured by Toray Industries, Inc.) with a nominal pore size of 0.3 μm was installed in the circulation line to prevent inflow of suspended particles to the feed tank. The temperature of the feed solution in the feed tank was controlled to maintain a set value by a heater in the tank and a heat exchanger installed in the circulation line. The feed solution in the feed tank was fed in series to Cells A and B, which were equipped with RO membranes, by a high-pressure pump. By adjusting three valves, namely, (1) the inlet valve to Cell A at the outlet of the high-pressure pump, (2) the bypass valve that returns the feed water from the high-pressure pump to the feed tank, and (3) the outlet valve of Cell B, the feed water to Cell A was set to the desired pressure and flow rate (i.e., *Re*). The pressure at the inlet and outlet of Cells A and B was continuously recorded using a sensor installed in the pressure gauge. The permeate through the RO membranes in Cells A and B was collected in a beaker with a siphon, and the permeate flow rate was measured by weighing the unit time mass with an electronic balance (model CG-300, Shinko Denshi Co., Ltd., Tokyo, Japan) and automatically recorded by a personal computer. The concentrate from Cell B was measured by a rotameter flowmeter and the concentrate flow rate was measured by weighing the unit time mass in a beaker with a siphon using an electronic balance (model CG-1500, Shinko Denshi Co., Ltd.). To exclude the effect of seed crystals that may have been present due to precipitation, the experiment was conducted using a one-pass method, in which the feed solution was not circulated, and the concentrate was discharged outside the system.

### 2.5. Mass-Transfer Coefficient Measurement

#### 2.5.1. Previous Studies

**Figure 3:**
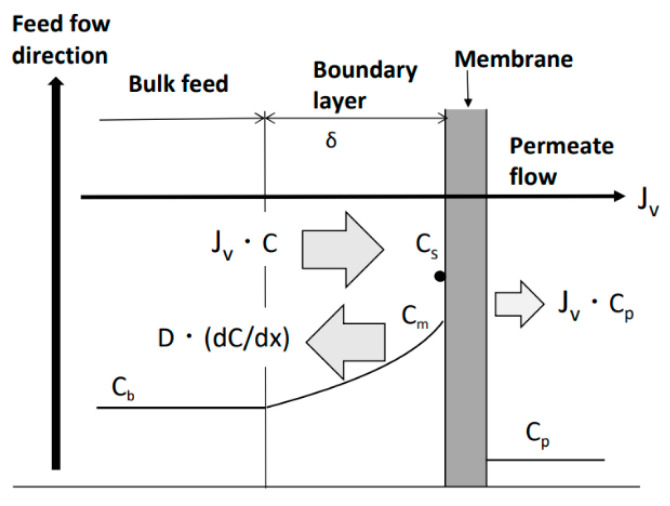
Concentration polarization phenomenon. *J_v_*: permeate flux; *J_v_C*: migration of solute with permeate flux; −*D*(d*C*/d*x*): diffusion of solute; *C_m_*: solute concentration on membrane surface; *C_s_*: saturated solute concentration; *C_b_*: solute concentration of bulk feed; *C_p_*: solute concentration of permeate flow; and *δ*: thickness of boundary layer.

Transport phenomena of the solute near the RO membrane surface are shown in Figure 3. To model these phenomena, the permeate flux equation based on concentration polarization is expressed as follows [62]:

*J_v_* = *k* ln{(*C_m_* − *C_p_*)/(*C_b_* − *C_p_*)}, (3)

where *J_v_*: permeate flux; *k*: mass-transfer coefficient; *C_b_*: bulk feed concentration; *C_p_*: permeate concentration; and *C_m_*: concentration at membrane surface.

From a chemical engineering point of view, the value of *k* is usually calculated using dimensionless correlation equations, such as the Sherwood (*Sh*), Reynolds (*Re*), or Schmidt (*Sc*) numbers, which are respectively expressed by the following equations:

*Sh* = *kd_h_*/*D*; (4)

*Re* = *ρud_h_*/*μ*; (5)

*Sc* = *μ*/(*ρD*), (6)

where *d_h_*: hydraulic diameter; *D*: diffusion coefficient; *ρ*: density of solution; *u*: feed flow velocity; and *μ*: viscosity of solution. The Leveque equation provides a dimensionless correlation for laminar flow in a channel [62]:

*Sh* = 1.86(*Re*·*Sc*·*d_h_*/*L*)^0.33^, (7)

where *L*: length of channel. The value of *k* is obtained using the Leveque equation for laminar flow in a channel (Equation (7)) and the Sherwood number (Equation (4)).

#### 2.5.2. Measurement of Mass-Transfer Coefficient by Velocity Variation Method

The flat membrane cell used in this work had a complicated shape in which the feed inlet and concentrate outlet were not at the edge of the cell, so the mass-transfer coefficient was directly measured using the velocity variation method [63,64]. The observed rejection (*R_obs_*) by actual measurement of the RO membrane is given by the following equation:

*R_obs_* = 1 − *C_p_*/*C_b_*. (8)

The real rejection (*R*) of the RO membrane is given by:

*R* = 1 − *C_p_*/*C_m_*. (9)

Substituting Equations (8) and (9) into Equation (3) and transforming it yields:

ln{(1 − *R_obs_*)/*R_obs_*} = ln{(1 − *R*)/*R*} + *J_v_*/*k*. (10)

In general, *k* is a function of the feed flow velocity (*u*) and the following equation can be applied, where *a* and *b* are constants:

*k* = *bu^a^*. (11)

Substituting Equation (11) into Equation (10) gives:

ln{(1 − *R_obs_*)/*R_obs_*} = ln{(1 − *R*)/*R*} + (*J_v_*/*bu^a^*). (12)

This means that a linear plot of ln{(1 − *R_obs_*)/*R_obs_*} against *J_v_*/*u^a^* is obtained and the real rejection *R* is given by extrapolation to the ordinate. Using this value of *R*, *k* is calculated by Equation (10).

### 2.6. Scale Precipitation Experiments

Experiments were conducted to investigate the precipitation of CaSO_4_ on the RO membrane. The effects of feed pressure, solute CaSO_4_ concentration, temperature, and *Re* on scale precipitation and membrane performance were investigated. Three types of RO membranes were used: UTC-70UL, UTC-70, and UTC-80, which had high, medium, and low permeate fluxes, respectively.

## 3. Results and Discussion

### 3.1. Mass-Transfer Coefficient

#### 3.1.1. Experimental Measurement of Mass-Transfer Coefficient by Velocity Variation Method

**Figure 4:**
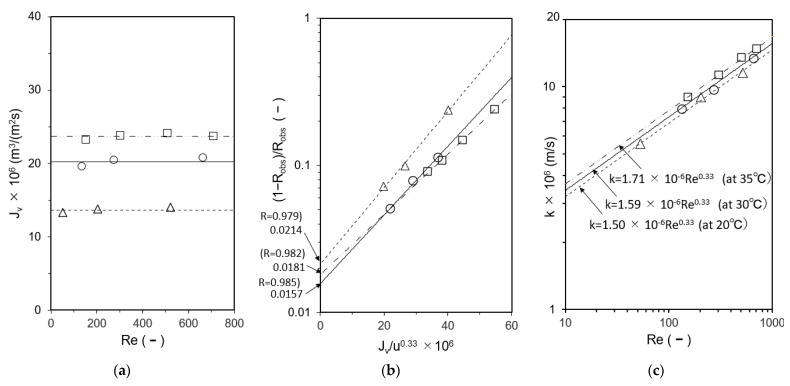
Determination of mass-transfer coefficients by velocity variation method. (**a**) Relationship between *Re* and *J_v_*, (**b**) Calculation of *R*, (**c**) Relationship between *Re* and *k*. Test conditions: solute: CaSO_4_; feed concentration: 140–160 mg/L; membrane: UTC-60; and temperature: Δ 20 °C, ○ 30 °C, and □ 35 °C.

Cells A and B were loaded with the UTC-60 low-rejection membrane. The results of measurements of *k* at 20 °C, 30 °C, and 35 °C as a function of Reynolds number are shown in Figure 4. The hydraulic diameter *d_h_* was calculated according to the method reported by Shock and Miquel [65]. Figure 4a shows the relationship between *Re* and *J_v_*, in which *J_v_* was constant and independent of *Re*, indicating that the experiment was conducted within the range where the velocity variation method can be applied. Figure 4b shows the relationship between *J_v_*/*u*^0.33^ and (1 − *R_obs_*)/*R_obs_*. The relationship between *Re* and *k* is shown in Figure 4c. The results agree well with those of Kimura and Sourirajan, who stated that *k* is proportional to *Re* to the power of 0.33 in the laminar region, confirming that the experiments were conducted in the laminar region [66]. Identical values of *k* were obtained for Cells A and B.

#### 3.1.2. Determination of Mass-Transfer Coefficient in Flat Membrane Cell

**Figure 5:**
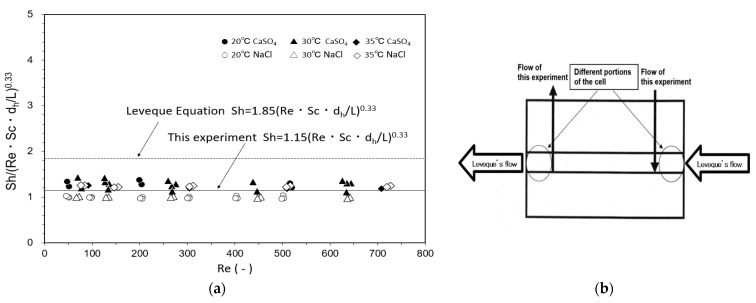
Comparison between experimental results and Leveque equation for determination of mass-transfer coefficient. (**a**) *Re* vs. *Sh*/(*Re Sc d_h_*/*L*)^0.33^, (**b**) Difference in flow in the cell between Leveque report and this experiment.

As described in Section 2.5.1, the Leveque equation, shown in Equation (7), is a dimensionless correlation equation for the mass-transfer coefficient in regions of laminar and channel flows. These experiments were conducted in the laminar and channel flow regions, as described in Section 3.1.1, so the experimental results for the CaSO_4_ and NaCl solutes at temperatures of 20 °C, 30 °C, and 35 °C were summarized in the form of the Leveque equation and plotted as shown in Figure 5a. Because the real rejection of CaSO_4_ was high in these experiments (approximately 98%), NaCl, which has a lower rejection of approximately 70%, was also examined. The concentrations of NaCl and CaSO_4_ in the feed solution were sufficiently low (100–160 mg/L) to assume that the physical properties of the feed solution, *ρ* and *μ*, were same as those of pure water. The values of *ρ* and *μ* of pure water at different temperatures are given by the Chemical Engineers’ Handbook [67]. The values of *D* of NaCl in water at different temperatures were given by their measured values and the equation for temperature dependence of the diffusion coefficient by Olson et al. [68]. The values of *D* of CaSO_4_ in water at different temperatures were given by Haskell’s equation [69] and the Chemical Engineers’ Handbook [67]. The results could be summarized by the following dimensionless correlation with a coefficient of 1.15:

*Sh* = 1.15(*Re*·*Sc*·*d_h_*/*L*)^0.33^. (13)

The difference between the coefficient of 1.86 of the Leveque equation and this experimental coefficient of 1.15 can be attributed to the difference in the geometry of the flat membrane cell used in the experiments, particularly the solution inlet and outlet positions, as shown in Figure 5b. Mass-transfer coefficients of the flat membrane cell under operating conditions could be calculated using Equations (4) and (13).

### 3.2. Permeate Flux Change with Time

#### 3.2.1. Introduction of Scaling-Based Flux Model

**Figure 6:**
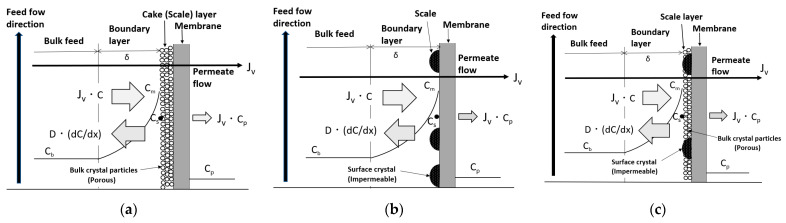
Scaling-based flux model. (**a**) Cake formation mechanism. (**b**) Surface blockage mechanism. (**c**) Mixed crystallization mechanism. *J_v_*: permeate flux; *J_v_C*: migration of solute with permeate flux; −*D*(d*C*/d*x*): diffusion of solute; *C_m_*: solute concentration on membrane surface; *C_s_*: saturated solute concentration; *C_b_*: solute concentration of bulk feed; *C_p_*: solute concentration of permeate flow; and *δ*: thickness of boundary layer.

Three types of scale precipitation mechanisms related to the decline in permeate flux in RO membranes are proposed: cake formation, surface blockage, and mixed crystallization, as shown in Figure 6 [27,28,31,33]. The convergent values of decline in permeate flux by each mechanism were studied using a newly proposed scaling-based flux model.

(a) Cake formation mechanism

Under the condition of no scale precipitation on the membrane surface, shown in Figure 3, Equation (3) is valid due to the concentration polarization in the region near the membrane surface. As shown in Figure 6a, when solute flux (*J_v_C*) increases, *C_m_* exceeds the saturated concentration of solute, *C_s_*, i.e., it becomes supersaturated. In supersaturated solutions, scale crystal nuclei are generated and grow into crystals. The crystals transfer to the membrane surface with *J_v_*, precipitate on the membrane surface, and form a cake (scale) layer. The permeate can pass through the gaps between the crystals of the cake layer. An increase in the thickness of the cake due to the precipitation of crystals on the membrane surface enlarges the permeation resistance of the cake; therefore, *J_v_* decreases, the amount of solute transferred to the membrane surface decreases, *C_m_* decreases, and the supersaturation and crystallization rate also decrease. Eventually, when *C_m_ = C_s_*, the crystal nuclei and crystals in solution near the membrane surface cease to form, and precipitation on the membrane surface also ceases. The increase in the thickness of the cake on the membrane surface also ceases, the permeation resistance becomes constant, and the permeate flux ceases to decrease and converges to a constant value.

(b) Surface blockage mechanism

As shown in Figure 6b, when *C_m_ > C_s_* due to concentration polarization, a crystal nucleus is generated and becomes a crystal on the membrane surface, which grows to cover the surface. The crystallized portion of the membrane surface blocks permeation, so the effective membrane area decreases, the permeate flux of the entire membrane surface decreases, and the amount of solute transferred to the membrane surface decreases. The solute concentrations of both the non-crystallized and crystallized parts of the membrane surface take on the same *C_m_* because the flow and diffusion rates of the solution are sufficiently large compared with the crystallization rate. The amount of solute transferred to the membrane surface decreases due to the decline in permeate flux. As a result, C_m_ decreases and the crystallization rate decreases. Eventually, when *C_m_ = C_s_*, the supply of solute to the membrane surface is in dynamic equilibrium with diffusion of solute back to the bulk, so crystal growth ceases and the area of crystal covering the membrane surface also ceases to increase. As a result, the permeable membrane area ceases to decrease and becomes constant, and the permeate flux of the entire membrane surface converges to a constant value.

(c) Mixed crystallization mechanism

Figure 6c shows the case where the cake formation and surface blockage mechanisms occur simultaneously when crystals are precipitated on the membrane surface. The concentration of the solute at the membrane surface, *C_m_*, becomes supersaturated due to concentration polarization and crystal nuclei are generated in the supersaturated solution, which grow into crystals. These are transferred by the permeate flux to precipitate on the membrane surface and form a cake (scale) layer. At the same time, crystal nuclei are generated on the membrane surface and become crystals that grow to cover the surface. Owing to both the increase in the permeation resistance of the permeable cake on the membrane surface and the decrease in the effective permeable membrane area caused by the growth of impermeable crystals, the permeate flux decreases and the amount of solute transferred to the membrane surface decreases. The difference in solute concentration between the surface of the impermeable crystals and that of the cake disappears because the flow and diffusion rates of the solution are sufficiently large compared with the crystallization rate, resulting in the same solute concentration, *C_m_*, on the surfaces of the impermeable crystals and the cake. As the amount of solute supplied to the membrane surface decreases, the solute concentration in the supersaturated solution and crystallization rate also decrease. Eventually, when *C_m_ = C_s_*, the generation of crystal nuclei and crystal growth in the solution near the membrane surface cease, and the precipitation of crystals on the membrane surface also ceases. At the same time, the growth of crystals on the membrane surface ceases, so the area of crystals covering the membrane surface ceases to increase. The permeable membrane area stops decreasing and becomes constant; therefore, the permeation resistance and permeable membrane area also become constant, and the permeate flux of the entire membrane surface converges to a constant value.

Although the trajectory of the permeate flux decline and the amount of scale on the membrane surface differ for the three mechanisms described above, both the precipitation and growth of crystals on the membrane surface cease when *C_m_* in the supersaturated state decreases and becomes equal to *C_s_*. In these three cases, the permeate flux *J_v_* of the entire membrane surface is considered to converge to a scaling-based critical flux (*J_sc_*), defined by:

*J_sc_* = *k* ln{(*C_s_* − *C_p_*)/(*C_b_* − *C_p_*)} = constant. (14)

*J_sc_* is obtained by substituting *k*, *C_b_*, and *C_p_*, which are obtained experimentally, and *C_s_* which are 2050, 2080, 2090 and 2100 mg/L at 20 °C, 25 °C, 30 °C and 35 °C, given by Chemical Handbook [70,71] into Equation (14). This concept is defined as the *scaling-based flux model*.

#### 3.2.2. Scaling Index

The scaling index (SCI) is defined as a discriminant equation for scale precipitation operating conditions as follows:

SCI = *J_v_*/*J_sc_* = *J_v_*/[*k* ln{(*C_s_* − *C_p_*)/(*C_b_* − *C_p_*)}]. (15)

(1) When SCI > 1, the following equation pertains from Equations (3) and (15):

ln{(*C_m_* − *C_p_*)/(*C_b_* − *C_p_*)} > ln{(*C_s_* − *C_p_*)/(*C_b_* − *C_p_*)}. (16)

Given that the natural logarithm is a monotonically increasing function, for *x*, *y* > 0, *x > y* ⇔ ln(*x*) > ln(*y*). Therefore, if Equation (16) is satisfied, the following equation is also satisfied:

(*C_m_* − *C_p_*)/(*C_b_* − *C_p_*) > (*C_s_* − *C_p_*)/(*C_b_* − *C_p_*). (17)

Rearranging the above gives the following equation:

*C_m_* > *C_s_*. (18)

In other words, when SCI > 1, then *C_m_ > C_s_*, so *C_m_* becomes larger than *C_s_* and scale precipitation on the RO membrane surface occurs.

(2) When SCI ≤ 1, the same consideration as above results in *C_m_ ≤ C_s_*, where *C_m_* is equal to or smaller than C_s_ and scale precipitation on the RO membrane surface does not occur.

#### 3.2.3. Osmotic Pressure Model

It is widely accepted that Equations (19) and (20) of the osmotic pressure model [62] can be applied to *J_v_* in the region where solute precipitation on the RO membrane surface does not occur:

*J_v_* = *A* (Δ*P* − Δ*π*); (19)

Δ*π* = *π*(*C_m_*) − *π*(*C_p_*), (20)

where *A*: pure water permeability; Δ*P*: operating pressure difference; Δ*π*: osmotic pressure difference; *π*(*C_m_*): osmotic pressure on membrane surface; and *π*(*C_p_*): osmotic pressure of permeate solution. Here, *C_m_* is given by the following equation, which is a variation of Equation (3):

*C_m_* = *C_p_* + (*C_b_* − *C_p_*) exp(*J_v_*/*k*). (21)

From Equations (19) and (20), *J_v_* is determined by *A*, Δ*P*, and Δ*π*. The *A* value of Equation (19) in this experiment was obtained directly from a pure water permeation experiment for each membrane.

#### 3.2.4. Experimental Results of Permeate Flux Change with Time

**Figure 7:**
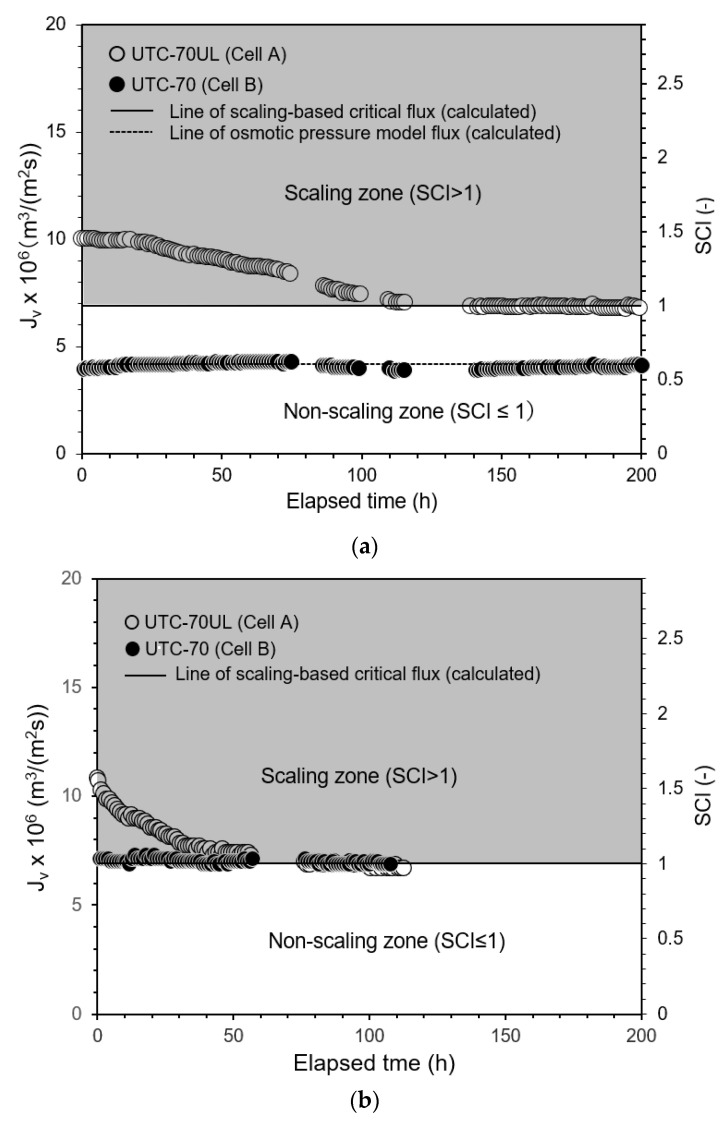

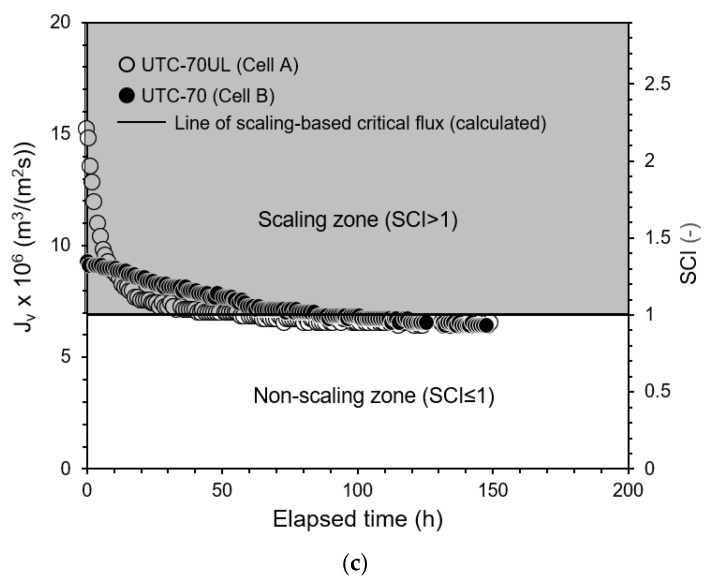
Time course of permeate flux and scaling-based critical flux. (**a**) Pressure: 0.5 MPa, (**b**) Pressure: 0.8 MPa, (**c**) Pressure 1.0 MPa. Test conditions: temperature: 30 °C; pressure: (**a**) 0.50 MPa, (**b**) 0.8 MPa, (**c**) 1.0 MPa; feed CaSO_4_ concentration: 875 mg/L; brine flow rate: 1.67 × 10^−6^ m^3^/s (= 100 mL/min); *Re*: (**a**) 125 (Cell A) and 123 (Cell B), (**b**) 127 (Cell A) and 123 (Cell B), (**c**) 127 (Cell A) and 124 (Cell B); and membrane: UTC-70UL (Cell A) and UTC-70 (Cell B). Scaling-based critical flux was *J_sc_* = *k* ln{(*C_s_* − *C_p_*)/(*C_b_* − *C_p_*)} = 6.9 × 10^−6^ m^3^/(m^2^ s). Osmotic pressure model flux in (**a**) was *J_v_* = *A* (Δ*P* − Δ*π*) = 4.22 × 10^−6^ m^3^/(m^2^ s) for UTC-70.

To analyze the mechanism of scale precipitation, the change in the permeate flux of the RO membrane with time due to scale precipitation was measured. The results are shown in Figure 7a. The experimental conditions were as follows: temperature: 30 °C; operating pressure: 0.50 MPa; CaSO_4_ concentration at inlet of Cell A: 875 mg/L; and brine flow rate at outlet of Cell B: 1.67 × 10^−6^ m^3^/s (= 100 mL/min). In Cell A, which was equipped with the high-permeate flux UTC-70UL RO membrane, the permeate flux decreased after the start of operation and converged to the scaling-based critical flux expressed by Equation (22):

*J_sc_* = *k* ln{(*C_s_* − *C_p_*)/(*C_b_* − *C_p_*)} = 6.88 × 10^−6^ m^3^/(m^2^ s). (22)

The following values for Cell A, given from experiments and calculations, were applied:

*Re* = 125; (23)

*k* = 1.59 × 10^−6^
*Re*^0.33^ m/s = 7.82 × 10^−6^ m/s; (24)

*C_s_* = 2090 mg/L; (25)

*C_b_* = 885 mg/L; (26)

*C_p_* = 29.9 mg/L. (27)

When *J_v_* converges to *J_sc_*, then *J_v_* = *J_sc_* and SCI = 1. Figure 7a shows the elapsed time on the abscissa, permeate flux on the first ordinate, and SCI on the second ordinate. The gray zone with SCI > 1, i.e., *J_v_* > *J_sc_*, is the scaling zone; the white zone with SCI ≤ 1, i.e., *J_v_* ≤ *J_sc_*, is the non-scaling zone.

Cell B was equipped with the medium-permeate flux UTC-70 RO membrane. The initial permeate flux (*J_v,_*_0_) was in the range of *J_v_*_,0_ ≤ *J_sc_*, i.e., the non-scaling zone of SCI ≤ 1. There was no precipitation of solute CaSO_4_ and the permeate flux maintained the initial value, which was consistent with the osmotic pressure model of Equation (28):

*J_v_* = *A*(Δ*P* − Δ*π*) = 4.22 × 10^−6^ m^3^/(m^2^ s). (28)

The following values for Cell B, given by experiments and calculations, were applied:

*A* = 9.52 × 10^−6^ m^3^/(m^2^ s MPa) (29)

Δ*P* = 0.50 MPa; (30)

*C_b_* = 896 mg/L; (31)

*C_p_* = 9.3 mg/L; (32)

*Re* = 123; (33)

*k* = 1.59 × 10^−6^
*Re*^0.33^ m/s = 7.78 × 10^−6^ m/s; (34)

*C_m_* = *C_p_* + (*C_b_* − *C_p_*) exp(*J_v_*/*k*) = 1540 mg/L; (35)

*π*(*C_m_*) = 0.0569 MPa; (36)

*π*(*C_p_*) = 0.0003 MPa. (37)

The value of *π* was given from the solute concentration and the van’t Hoff equation [72]:

Δ*π* = *π*(*C*_m_) − *π*(*C*_p_) = 0.0566 MPa. (38)

The results when the pressure was set at 0.8 MPa are shown in Figure 7b and when 1.0 MPa, in Figure 7c.

### 3.3. Study of Convergent Value of Permeate Flux with Scale Precipitation

#### 3.3.1. Effect of Operating Pressure on Convergent Value of Permeate Flux

**Figure 8:**
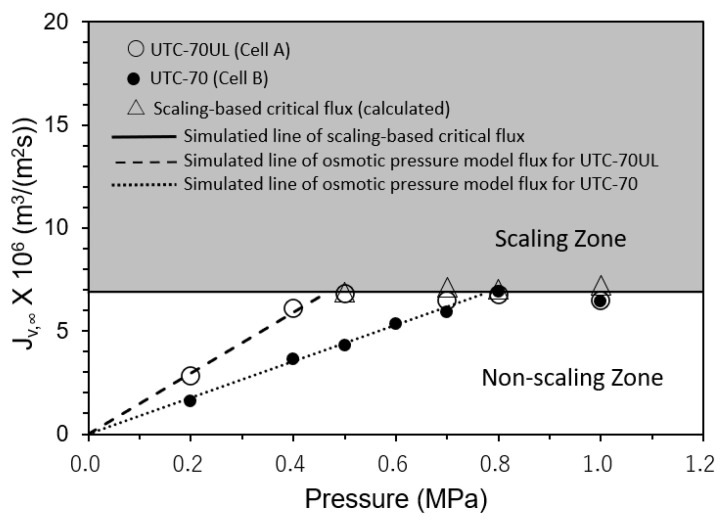
Effect of operating pressure on convergent permeate flux. Test conditions: temperature: 30 °C; feed CaSO_4_ concentration: 875 mg/L; brine flow rate: 1.67 × 10^−6^ m^3^/s (= 100 mL/min); *Re*: 124–127 (Cell A) and 122–124 (Cell B); and membrane: UTC-70UL (Cell A) and UTC-70 (Cell B). Simulated line of scaling-based critical flux is given by *J_sc_* = *k* ln{(*C_s_* − *C_p_*)/(*C_b_* − *C_p_*)} = 6.9 × 10^−6^ m^3^/(m^2^ s). Simulated lines of osmotic pressure model flux are given by *J_v_* = *A* (Δ*P* − Δ*π*), *A* = 1.80 × 10^−5^ m^3^/(m^2^ s) for UTC-70UL and *J_v_* = *A*(Δ*P* − Δ*π*), *A* = 9.52 × 10^−6^ m^3^/(m^2^ s) for UTC-70.

The relationships between the operating pressures and convergent values of the permeate fluxes (*J_v,_*_∞_) were measured. The results are shown in Figure 8. Cell A was equipped with UTC-70UL, a high-permeate flux membrane, and Cell B with UTC-70, a medium-permeate flux membrane. In both Cells A and B, scale precipitation did not occur in the non-scaling zone where the initial permeate flux (*J_v,_*_0_) was in the range of *J_v,_*_0_ ≤ *J_sc_*, i.e., SCI ≤ 1. The permeate flux was proportional to the operating pressure and consistent with the osmotic pressure model flux with the pure water permeability coefficients of UTC-70UL and UTC-70, as shown in Figure 8.

In the scaling zone, where *J_v_*_,0_ was in the range of *J_v,_*_0_ > *J_sc_*, i.e., SCI > 1, scale precipitated on the membrane surface, and the permeate flux decreased with time and converged to the calculated scaling-based critical flux (*J_sc_*) (triangle marks in Figure 8). The four values of *J_sc_* were almost same and the average values were as follows:

*J_sc_* = *k* ln(*C_s_* − *C_p_*)/(*C_b_* − *C_p_*)} = 6.9 × 10^−6^ m^3^/(m^2^ s). (39)

*J_sc_* was constant and independent of the operating pressure. Equation (39) is in good agreement with the experimental results.

#### 3.3.2. Effect of CaSO_4_ Concentration on Convergent Value of Permeate Flux

**Figure 9:**
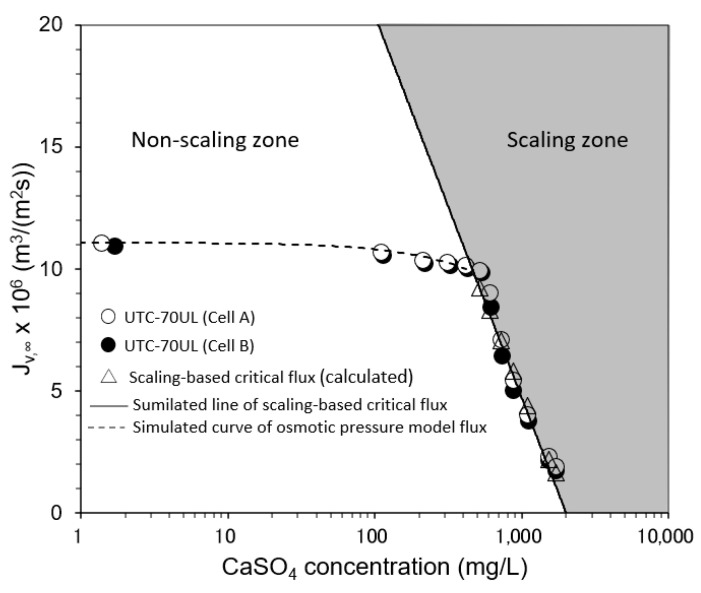
Effect of CaSO_4_ concentration on convergent permeate flux. Test conditions: temperature: 20 °C; pressure: 0.80 MPa; brine flow rate: 1.67 × 10^−6^ m^3^/s (= 100 mL/min); *Re*: 93–98 (Cell A) and 92–94 (Cell B); and membrane: UTC-70UL (Cells A and B). Simulated line of scaling-based critical flux is given by *J_sc_* = *k* ln{(*C_s_* − *C_p_*)/(*C_b_* − *C_p_*)} = (5.17 × 10^−5^ − 6.79 × 10^−6^ ln *C_b_*) m^3^/(m^2^ s). Simulated line of osmotic pressure model flux is given by *J_v_* = *A* (Δ*P* − Δ*π*), *A* = 1.41 × 10^−5^ m^3^/(m^2^ s) for UTC-70UL.

The relationship between the CaSO_4_ concentration and the convergent value of permeate flux is shown in Figure 9. In the non-scaling zone, where the CaSO_4_ concentration was small and the initial permeate flux was in the range of *J_v,_*_0_ ≤ *J*_sc_, i.e., SCI ≤ 1, there was no decrease in permeate flux with time and *J_v_* was consistent with the osmotic pressure model flux. In the scaling zone, where the initial flux was in the range of *J_v,_*_0_ > *J_sc_*, i.e., SCI > 1, the permeate flux decreased with time and converged to the calculated scaling-based critical flux (triangle marks in Figure 9). *C_p_* was small enough compared with *C_b_* to be ignored, so *J_sc_* could be expressed as a function of *C_b_* as follows:

*J_sc_* = *k* ln(*C*_s_ − *C_p_*)/(*C_b_* − *C_p_*)} = (5.17 × 10^−5^ − 6.79 × 10^−6^ ln *C_b_*) m^3^/(m^2^ s), (40)

where

*k* = 1.50 × 10^−6^
*Re*^0.33^ m/s = 6.79 × 10^−6^ m/s (at 20 °C, *Re* = 97). (41)

Equation (40) is in good agreement with the experimental results.

#### 3.3.3. Effect of Temperature on Convergent Value of Permeate Flux

**Figure 10:**
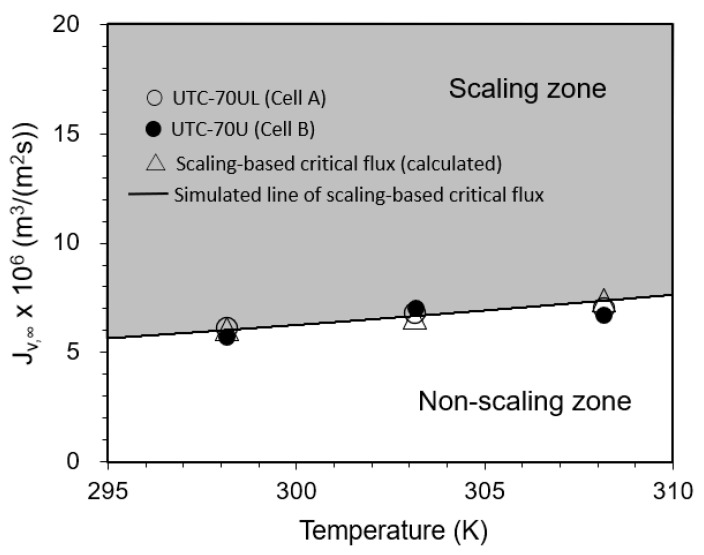
Effect of temperature on convergent permeate flux. Test conditions: pressure: 0.80 MPa; feed CaSO_4_ concentration: 875 mg/L; brine flow rate: 1.67 × 10^−6^ m^3^/s (= 100 mL/min); *Re*: 110 (298 K), 126 (303 K), 133 (308 K) for Cell A and 108 (298 K), 123 (303 K), 128 (308 K) for Cell B; and membrane: UTC-70UL (Cells A and B). Simulated line of scaling-based critical flux is given by *J_sc_* = *k* ln{(*C_s_* − *C_p_*)/(*C_b_* − *C_p_*)} = (1.23 × 10^−5^ (*T* exp(−2005/*T*))^0.67^) m^3^/(m^2^ s).

The relationship between temperature and the convergent value of the permeate flux is shown in Figure 10. Under the experimental conditions, the initial permeate flux was in the region of the scaling zone in the range of *J_v,_*_0_ > *J_sc_*, i.e., SCI > 1. From the beginning of operation, the permeate flux decreased with time and converged to the calculated value of the scaling-based critical flux (triangle marks in Figure 10).

Substituting Equations (4)–(6) into Equation (13), the relationship between the mass-transfer coefficient (*k*) and diffusion coefficient (*D*) of the temperature-dependent term can be summarized as follows:

*k = αD*^0.67^, (42)

where

*α =* 1.15(*u*/*L*)^0.33^(1/*d_h_*)^0.34^. (43)

The relationship between *D* and *T* can be expressed as follows from Wilke’s equation [67,73]:

*Dμ*/*T* = *c* (constant). (44)

The relationship between *μ* and *T* was expressed by Andrade as follows [74]:

*μ* = *a*_1_ exp(*b*_1_/*T*), (45)

where *a*_1_ and *b*_1_ are material constants. From Equations (44) and (45), the relationship between *D* and *T* is expressed by the following equation:

*D* = (*c*/*a*_1_)(*T* exp(−*b*_1_/*T*)). (46)

Substituting Haskell’s equation, data from the Chemical Engineers’ Handbook, and experimental data yields the following equation [67,69]:

*k* = 1.38 × 10^−5^(*T* exp(−2005/*T*))^0.67^ m/s. (47)

The simulated line of *J_sc_* with respect to *T* is given as follows:

J_sc_ = k ln{(C_s_ − C_p_)/(C_b_ − C_p_)} = 1.23 × 10^−5^(T exp(−2005/T))^0.67^ m^3^/(m^2^s). (48)

Equation (48) is in good agreement with the experimental results.

#### 3.3.4. Effect of Reynolds Number on Convergent Value of Permeate Flux

**Figure 11:**
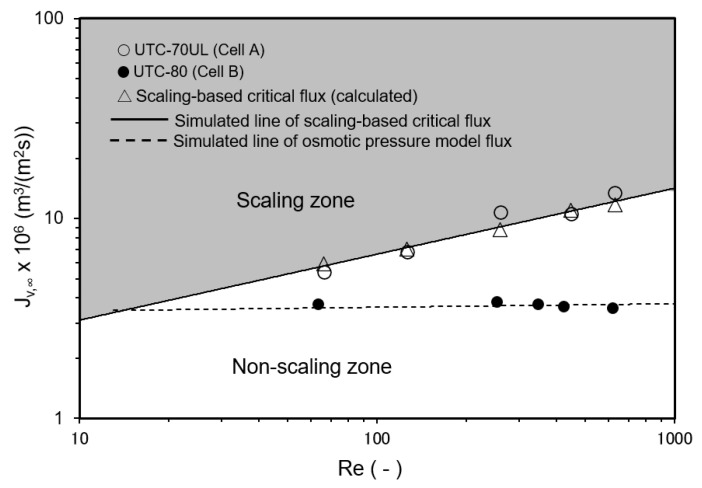
Effect of Reynolds number on convergent permeate flux. Test conditions: temperature: 30 °C; pressure: 0.80 MPa; feed CaSO_4_ concentration: 875 mg/L; and membrane: UTC-70UL (Cell A) and UTC-80 (Cell B). Simulated line of scaling-based critical flux is given by *J_sc_* = *k* ln{(*C_s_* − *C_p_*)/(*C_b_* − *C_p_*)} = 1.45 × 10^−6^ *Re*^0.33^ m^3^/(m^2^ s). Simulated line of osmotic pressure model flux was *J_v_* = *A* (Δ*P* − Δ*π*), *A* = 4.83 × 10^−6^ m^3^/(m^2^ s MPa) for UTC-80.

The relationship between *Re* and the convergent value of the permeate flux is shown in Figure 11. Cell A was equipped with the UTC-70UL membrane, which had a high permeate flux, and the operating conditions shown in Figure 11 were in the scaling zone where *J_v,_*_0_ > *J_sc_*, i.e., SCI > 1. The flux decreased with time and converged to the calculated scaling-based critical flux (triangle marks in Figure 11). *J_sc_* can be expressed as a function of *Re* as follows:

*J_sc_* = *k* ln{(*C_s_* − *C_p_*)/(*C_b_* − *C_p_*)} = 1.45 × 10^−6^ *Re*^0.33^ m^3^/(m^2^ s). (49)

Equation (49) is good agreement with the experimental results.

Cell B was equipped with a UTC-80 membrane for seawater desalination, which had a low permeate flux, and the operating conditions were in the non-scaling zone where *J_v,_*_0_ ≤ *J_sc_*, i.e., SCI ≤ 1. Scale precipitation on the membrane did not occur and the permeate flux was consistent with the osmotic pressure model flux (Equation (19)).

## 4. Advice for Reverse Osmosis Plant Designers, Operators, and Membrane Manufacturers

To prevent scale precipitation in RO plants, it is recommended to calculate the scaling index (SCI) for solutes that may precipitate, such as CaSO_4_, CaCO_3_, and SiO_2_, in the most downstream RO module of an RO plant. Scale precipitation on the RO membranes can then be prevented by designing the plant and setting the operating conditions so that SCI ≤ 1, which is in the non-scaling zone.

For practical use, the modified scaling-based critical flux *J’_sc_* = *k* ln(*C_s_*/*C_b_*) can be used instead of *J_sc_* in the region of high observed rejection of the RO membrane, and *J*’*_sc_* can be calculated regardless of membrane type if the value of *k* of the RO module is known. For the experiments shown in Figure 8, Figure 9, Figure 10 and Figure 11, the errors of *J*’*_sc_* with respect to *J_sc_* were less than 7% when the observed rejection of the RO membrane was greater than 90%; for the observed rejections of 88.6%, 81.1%, and 66.6%, the errors of *J*’*_sc_* with respect to *J_sc_* were 7.7%, 18.2%, and 35.3%, respectively. Therefore, the modified scaling-based critical flux (*J*’_sc_) should be carefully applied to practical operation, considering whether the observed rejection of the RO membrane is high enough.

It is recommended that RO membrane manufacturers measure the mass-transfer coefficient (*k*) of commercially available RO modules with the velocity variation method and disclose it to users, because this can be more easily measured by the manufacturer than by individual users. This will allow RO plant designers and operators to easily calculate the scaling-based critical flux and SCI, and to design and operate the RO plant to avoid scale precipitation.

## 5. Conclusions

1. The phenomena of scale precipitation on a membrane surface were investigated. Using a newly proposed scaling-based flux model, the permeate fluxes of three mechanisms related to scale precipitation phenomena—cake formation, surface blockage, and mixed crystallization—were explained to converge to the same scaling-based critical flux, defined by Equation (14).
2. The scaling-based critical flux and scaling index (SCI) defined by Equation (15) were investigated. An operating condition described by SCI > 1 is a scaling zone, in which scale precipitates on the RO membrane surface, and the permeate flux decreases with time and finally converges to the scaling-based critical flux. An operating condition described by SCI ≤ 1 is a non-scaling zone, in which there is no scale precipitation on the RO membrane surface and no decline in permeate flux with time.
3. Experiments were conducted to investigate the effects of operating conditions, such as pressure, solute concentration, temperature, and *Re*. It was found that scale precipitation on the RO membrane surface could be determined by the scaling index (SCI). In the non-scaling zone (SCI ≤ 1), there was no scale precipitation on the membrane surface and the permeate flux was constant with time; in the scaling zone (SCI > 1), the permeate flux decreased with time and finally converged to the scaling-based critical flux.

## Figures and Tables

**Table 1:** Permeate flux and salt rejection of reverse osmosis membranes used in this study.

	Membrane Model			
	UTC-60	UTC-70UL	UTC-70	UTC-80
Performance				
Permeate flux (m^3^/(m^2^d)	0.78	1.15	1.26	0.65
Salt rejection (%)	70.9	99.5	99.7	99.9
Test conditions				
Applied pressure (MPa)	0.35	0.75	1.5	5.5
Temperature (°C)	25	25	25	25
NaCl concentration (mg/L)	500	1500	1500	3.5% Seawater
pH (−)	6.5	6.5	6.5	6.5

## Data Availability

The data presented in this study are available from the corresponding author on reasonable request.

## Abbreviations

*A*	Pure water permeability (m^3^/(m^2^ s Pa))
*a*	Constant
*a*_1_	Constant
*b*	Constant
*b*_1_	Constant
*C*	Solute concentration (mg/L)
*C_b_*	Solute concentration of bulk feed (mg/L)
*C_m_*	Solute concentration on membrane (mg/L)
*C_p_*	Solute concentration of permeate (mg/L)
*C_s_*	Saturated concentration of solute (mg/L)
*c*	Constant
*D*	Diffusion coefficient (m^2^/s)
*d_h_*	Hydraulic diameter (m)
*J_sc_*	Scaling-based critical flux (m^3^/(m^2^ s))
*J’_sc_*	Modified scaling-based critical flux (m^3^/(m^2^ s))
*J_v_*	Permeate volume flux (m^3^/(m^2^ s))
*J_v,_*_0_	Initial permeate volume flux (m^3^/(m^2^ s))
*J_v,∞_*	Convergent permeate volume flux (m^3^/(m^2^ s))
*k*	Mass-transfer coefficient (m/s)
*L*	Length of flow channel of cell (m)
*P*	Operating pressure (MPa)
Δ*P*	Operating pressure difference (MPa)
*R*	Real rejection (−)
*R_obs_*	Observed rejection (−)
*Re*	Reynolds number (−)
*Sc*	Schmidt number (−)
*Sh*	Sherwood number (−)
*SCI*	Scaling index (−)
*T*	Temperature (K)
*u*	Flow velocity (m/s)
*x*	Distance (m)
*α*	Constant
*δ*	Boundary layer thickness (m)
*μ*	Viscosity (Pa s)
*π*	Osmotic pressure (MPa)
Δ*π*	Osmotic pressure difference (MPa)
*ρ*	Density (kg/m^3^)
